# Noble Metal Nanomaterial-Based Biosensors for Electrochemical and Optical Detection of Viruses Causing Respiratory Illnesses

**DOI:** 10.3389/fchem.2021.672739

**Published:** 2021-05-13

**Authors:** Hye Kyu Choi, Myeong-Jun Lee, Sang Nam Lee, Tae-Hyung Kim, Byung-Keun Oh

**Affiliations:** ^1^Department of Chemical and Biomolecular Engineering, Sogang University, Seoul, South Korea; ^2^Uniance Gene Inc., Seoul, South Korea; ^3^School Integrative Engineering, Chung-Ang University, Seoul, South Korea

**Keywords:** noble metal nanomaterials, virus detection, electrochemical biosensor, optical biosensor, COVID-19

## Abstract

Noble metal nanomaterials, such as gold, silver, and platinum, have been studied extensively in broad scientific fields because of their unique properties, including superior conductivity, plasmonic property, and biocompatibility. Due to their unique properties, researchers have used them to fabricate biosensors. Recently, biosensors for detecting respiratory illness-inducing viruses have gained attention after the global outbreak of coronavirus disease (COVID-19). In this mini-review, we discuss noble metal nanomaterials and associated biosensors for detecting respiratory illness-causing viruses, including SARS-CoV-2, using electrochemical and optical detection techniques. this review will provide interdisciplinary knowledge about the application of noble metal nanomaterials to the biomedical field.

## Introduction

Viruses are infectious pathogens that require hosts for parasitic entry and cause significant disease due to their properties of propagation and genetic replication (Mokhtarzadeh et al., 2017; Abid et al., 2021). Severe acute respiratory syndrome coronavirus (SARS-CoV-2), which causes COVID-19, is raging rapidly worldwide because of the pandemic. Respiratory illness-inducing viruses, such as SARS-CoV-2, influenza virus, and Middle East respiratory syndrome coronavirus (MERS-CoV), have gained tremendous scientific interest due to their strong viability, transmissibility, and fatalities associated with their infections. In biomedical fields, numerous studies have been performed to diagnose and treat diseases caused by these viruses.

However, although treatments for previously existing virus infections have been developed, there remain obstacles to the rapid development of specific drugs or vaccines that respond perfectly to variants of respiratory illness-inducing viruses or newly discovered viruses (Srivastava et al., 2021). Therefore, biosensing techniques are an interesting and suitable option for exploring treatments of such infections; these treatments are aided by detecting the target virus for early diagnosis of the pathogenic disease (Afsahi et al., 2018; Dronina et al., 2021).

A biosensor is a medical platform to detect environmental components or biocomponents using numerous techniques, such as electrochemical detection using redox properties of materials, fluorescence detection with fluorescent dyes, and surface-enhanced Raman scattering (SERS) by the unique optical properties of materials (Goode et al., 2015). For early detection of the virus, several targets such as nucleic acids and proteins are used from biofluids (Lee et al., 2018). Furthermore, to enhance the selectivity and sensitivity of virus detection, various nanomaterials are used because of their small size, increased surface area, conductivity, and optical characteristics. Noble metal nanomaterials (e.g., gold, silver, and platinum) have been widely used in biosensors because of their high conductivity, biocompatibility, and stability (Wang, 2012).

Furthermore, they enable target virus detection directly or indirectly with a rapid response. The excellent stability of noble metal nanomaterials ensures the reproducibility of virus detection results using biosensors. With these advantages, biosensors comprising noble metal nanomaterials have been used as a biosensing platform with high performance, including high selectivity and sensitivity. In this mini-review, we focus on noble metal nanomaterials that exhibit suitable electrical and optical properties and provide detailed information on electrochemical and optical biosensors comprising noble metal nanomaterials aimed at detecting respiratory illness-inducing viruses. recently developed novel biosensors for SARS-CoV-2 detection are explored in this mini-review. We expect this mini-review to present the broad prospects of the use of biosensors for the detection of respiratory illness-inducing viruses and assist researchers with an advanced diagnostic system to overcome the pandemic.

## Noble Metal Nanomaterials For Biosensors

Noble metals are metallic elements (e.g., ruthenium, rhodium, palladium, platinum, gold, and silver) with outstanding resistance to high temperatures and chemical reactions (Hämäläinen et al., 2014; Sow et al., 2020). Noble metal nanomaterials have attracted scientific and technology research because of their small size (0.1–100 nm) and unique chemical and physical properties. For example, noble metal nanomaterials have been used as catalysts to reduce pollutants from exhausts because the surface of noble metal nanomaterials functions as the active sites for redox molecules and increases catalytic activities when the surface is in a zero-valent state (Hegde et al., 2009; Wang and Gu, 2015). Furthermore, noble metal nanomaterials have been used in biosensors because they can precisely and accurately detect target biocomponents because of their quantum mechanical properties due to their small size, biocompatibility, and easy accessibility for modification (Doria et al., 2012; Zhu et al., 2016; Craciun et al., 2017).

The gold nanoparticle (AuNP) is a noble metal nanomaterial studied for over 100 years (Giljohann et al., 2010). It has unique properties, including high electrical conductivity, biocompatibility, and stability (Sardar et al., 2009). Furthermore, surface modification of the gold surface can be easily achieved using a thiol group via the gold–thiol covalent attachment. These properties provide a convenient functionalization approach for AuNPs, particularly for their application in biomedical fields (Choi et al., 2020). In the electrochemical biosensing field, the fine electrical conductivity of AuNPs is useful when the target biomolecules are present in a small amount and the electrochemical signals from the redox reaction of biosensor samples are extremely small. Because of the excellent electrical conductivity of AuNPs, electrochemical biosensors comprising AuNPs have been developed by many researchers to achieve target biomolecule detection with high sensitivity and selectivity.

Furthermore, optical properties, such as surface plasmon resonance (SPR), differ depending on the structure of the AuNP. For example, Au nanorods, Au nanocages, and hollow Au nanospheres exhibit near-infrared absorption. In contrast, AuNPs absorb visible SPR, and the SPR property is controlled by the size of the AuNP (Guo and Wang, 2011). Given that optical biosensors comprise materials with novel and remarkable optical properties, AuNP is a promising material for use in optical biosensors.

Like AuNP, the silver nanoparticle (AgNP) is a suitable candidate for biosensors. Silver is a noble metal nanomaterial with its first recorded medical use dating back to the eighth century (Ravindran et al., 2013). The prior property of the silver is an SPR property that can be applied to photonic and sensor applications. Furthermore, the optoelectronic properties of silver can be optimized for the size, shape, and composition of AgNP because the optical properties originate from the collective oscillations of electrons. Therefore, AgNPs have been modified into various forms and compositions depending on the purpose of their application.

For example, Liu et al. synthesized a sliver nanocluster (AgNC) for label-free DNA detection (Liu et al., 2014). In this study, two types of AgNCs were used as fluorophores because the fluorescent properties of AgNCs were primarily sequence- and structure-dependent. Each type of the AgNCs was tailored to two different regions of a probe. Under the existence of target DNA, the two different AgNCs exhibited opposite fluorescence signals, such that the fluorescence signal from one AgNC increased when that from the other AgNC decreased. Finally, the ratio of the two different fluorescence signals could be used to detect the target DNA with high sensitivity. Moreover, an Ag nanorod with an aligned array was used to enhance the SERS signals (Chaney et al., 2005). The optimal aspect ratio of the Ag nanorod and arrangement of the nanoarray could successfully enhance the signals.

Furthermore, numerous electrochemical biosensors use AgNPs as a core component to enhance the detection performance due to their high electrical conductivity (Shin et al., 2017; Xu et al., 2019). However, there remains a disadvantage to using AgNPs for *in vivo* applications because the high densities of AgNPs exhibited toxicity during oxidation. Desireddy et al. synthesized ultrastable AgNPs to enhance the biocompatibility of AgNPs (Desireddy et al., 2013). The researchers developed a new approach to synthesizing ultrastable AgNPs involving the use of *p*-mercaptobenzoic acid to protect the ligand shell. The synthesized ultrastable AgNPs demonstrated excellent stability due to the Ag_2_S_5_ capping structure, a silver thiolate protecting layer. As described, AgNPs could be a novel component for various biomedical applications, including biosensors, because of their fascinating optical and electrochemical properties and biocompatibility if they are modified or capsulated with stable materials.

Platinum (Pt) has gained interest as an excellent catalyst because of its high stability in acid electrolytes and remarkable catalytic property for hydrogen redox reactions. In addition to the high cost associated with its rarity in nature, Pt—a small particle that is not a bulk-form—is conventionally used for chemical and biological applications. The Pt nanoparticles (PtNPs) for the catalytic application are from 2 to 5 nm, and the catalytic activity is particle-size dependent (Kinoshita, 1990). The excellent catalytic property of PtNPs can support electrochemical biosensors that use high oxygen redox activity (Garlyyev et al., 2019).

Accordingly, researchers have studied and developed various types of PtNPs and synthetic methods for controlling the shape of PtNPs to use them in specific applications like biosensors. For instance, Inaba et al. controlled the growth and shape formation of PtNPs to enhance their electrochemical properties (Inaba et al., 2006). In their research, cubic PtNPs were synthesized from K_2_PtCl_4_ solution with a sodium polyacrylate. Depending on the reaction temperature, the molecular weight of polyacrylate, and the Pt/polyacrylate ratio, the shape and size of PtNPs were modified, affecting the electrochemical properties.

Furthermore, spherical, nanorod, and nanowire form of Pt could be synthesized using preparative conditions (Ramirez et al., 2007). Because the catalytic properties of PtNPs could be changed by optimizing their shape and size, various types of PtNPs are desirable for use in electrochemical biosensors (Yang et al., 2006; Nguyen et al., 2019; Eom et al., 2020). Like other noble metal nanomaterials, the optical properties of PtNPs are also affected by their shape and size (Bigall et al., 2008). Accordingly, PtNPs have been used in optical biosensors (Tata et al., 2018; Cheng et al., 2019).

## Biosensors Composed of Noble Metal Nanomaterials for Detecting Virus Causing Respiratory Problems

Because of the unique properties of noble metal nanomaterials, they have been used in biosensors to detect viruses. Electrochemical and optical sensors are powerful analytical tools for real-time analysis of various samples (Majdinasab et al., 2019). The excellent properties of noble metal nanoparticles are suited for the design of optical and electrochemical biosensors, such that many researchers have developed noble metal nanoparticles and used them as an immobilization linker of bio-receptors, signal enhancement material, and signal transducers in biosensors for electrochemical and optical detection (Zhao X. et al., 2020). Recently, many biosensors for virus detection have been developed due to the coronavirus pandemic, and noble metal nanoparticles are at the center of these developments. In the following sections, we introduce various biosensors that use noble metal nanoparticles (AuNPs, AgNPs, and PtNPs) for detecting viruses that cause respiratory problems.

## Electrochemical Biosensors For Virus Detection

Electrochemical sensing technology has been widely used in biosensing due to its excellent properties of high sensitivity, speed, and simplicity (Khan et al., 2020). These technologies, such as cyclic voltammetry (CV), amperometry, and electrochemical impedance spectroscopy (EIS), can analyze electrochemical activities like the reduction and oxidation of biomolecules or chemicals (Cho et al., 2020; Noori et al., 2020). Because these sensing methods are based on electron transfer, it is essential to increase electron transfer rates and electrode surface area to enhance the electrochemical signal and increase sensitivity (Shin et al., 2020; Zhang L. Y. et al., 2020). In this context, the use of noble metal nanoparticles has received increasing attention in electrochemical biosensors for several reasons. The incorporation of noble metal nanoparticles in the biosensing platform can enhance the electrical response signal because of its attractive properties, such as high electrical conductivity, high surface area, and effectiveness in adhering to biomolecules (Jo et al., 2019; John et al., 2021).

**Figure 1:**
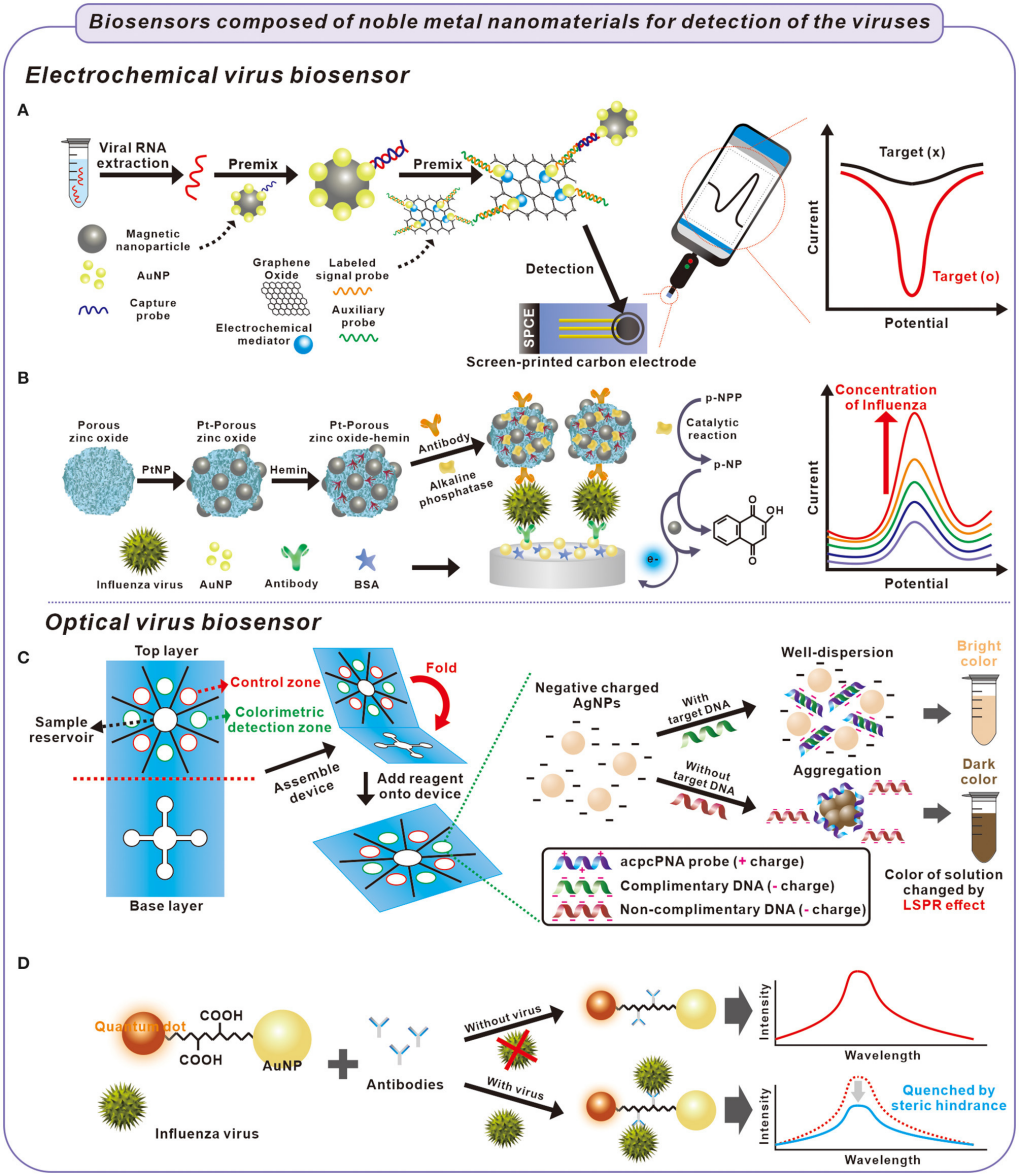
Fabrication process of electrochemical and optical biosensors for detection of viruses causing respiratory illness and principle of detection of the viruses. **(A)** Extracted viral RNA was captured with a capture probe on AuNPs and collected using magnetic nanoparticles. The viral RNA was electrochemically detected on a screen-printed carbon electrode composed of graphene oxide, mediator, and probes (Zhao et al., 2021). **(B)** Influenza virus was captured between antibodies in sandwich-structured biosensor composed of Pt-porous zinc oxide-hemin and AuNPs. The influenza virus was electrochemically detected through the catalytic oxidation of p-NP after catalytic reaction of p-NPP inside the Pt-porous-zinc oxide hemin structure (Yang et al., 2016). **(C)** The influenza virus was detected by the colorimetric biosensor. In the presence of target DNA from the influenza virus, negative charged AgNPs were well-dispersed because of the hybridization of probe and target DNA, with a bright color. In contrast, the AgNPs were aggregated with probe DNA in the absence of target DNA, which cause the solution color to darken (Teengam et al., 2017). **(D)** The influenza virus was optically detected using quenching effect. When the influenza virus was capture by the antibodies, the intensity of QDs immobilized AuNPs decreased by steric hindrance (Nasrin et al., 2020).

The effectiveness immobilization of biomolecules on noble metal nanoparticles is crucial in the electrochemical biosensor because the electrochemical signal is highly dependent on chemicals or biomolecules that function as transducers, signal-inducers, and capturing agents for target biomolecules (Zhang et al., 2020). the interaction between the thiol group of biomolecule and the surface of a noble metal nanoparticle have been widely used in electrochemical biosensors because of the effectiveness in adhering the target biomolecule to the surface of the nanoparticle. For example, Zhao et al. reported an ultrasensitive electrochemical detection technology using p-sulfocalix[8]arene functionalized reduced graphene oxide with AuNPs, toluidine blue, and the labeled signal probe (Au@SCX8-RGO-TB) for targeting the RNA of SARS-CoV-2 (Zhao et al., 2021). Through the coordination of AuNP-sulfhydryl groups, the signal probe and other materials were immobilized with AuNPs anchored on the material surface. The RGO-SCX8-Au and the Au@Fe_3_O_4_ probe constructed a super-sandwich structure using the viral RNA and probe's DNA fragments. The magnetism of the Au@Fe_3_O_4_ nanoparticle helped separate the super-sandwich structure from the solution. The separated Au@SCX8-RGO-TB via viral RNA was dropped on the carbon-three electrode screen-printing carbon electrode (SPCE). In Au@SCX8-RGO-TB, toluidine blue functioned as an electrochemical signal-inducing molecule. By analyzing the signal of separated Au@SCX8-RGO-TB using differential pulse voltammetry (DPV) which is one of the electrochemical detection methods, the amount of viral RNA can be analyzed (Figure 1A). This biosensor demonstrated high specificity and sensitivity, in which the limit of detection (LOD) of the clinical specimen was 200 copies/mL without reverse transcription and nucleic acid amplification.

AuNPs have the characteristic of electrostatic interactions or physisorption with a protein; therefore, they can be used to immobilize antibodies on the electrode. With these properties, a fluorine-doped tin oxide electrode with AuNPs and SARS-CoV antibody was fabricated. When the SARS-CoV spike protein was immobilized on the electrode by the antibody-antigen reaction, the peak current of the electrode was increased. Based on the results, the biosensor successfully detected SARS-CoV spike protein with LOD = 7.8 × 10^−1^ pg/mL using DPV (Mahari et al., 2020).

AgNPs interact with viruses, such as influenza. Through this well-investigated antiviral activity phenomenon, the interaction between AgNPs and viruses can be used for low-level detection of virus based on the inherent electrochemical activity of the virus–metal nanoparticle interaction in the solution (Galdiero et al., 2011). For example, Sepunaru et al. developed an influenza electrochemical biosensor using the interaction between AgNPs and the virus. Because of the oxidation of AgNPs, the AgNP-modified virus exhibited an enhanced electrochemical signal compared to the virus. With the AgNP-tagged virus, the electrochemical biosensor in their research can rapidly detect the influenza viruses at a single virus level (Sepunaru et al., 2016).

The high surface area of nanoparticles and conductivity of the metals themselves help increase electrochemical signals. Layqah and Eissa developed a MERS-CoV electrochemical immuno-sensor using an array of AuNP-modified carbon electrodes (Layqah and Eissa, 2019). In their research, AuNPs functioned as a signal enhancer by increasing the electrode surface area and electron transfer rate. With the square wave voltammetry (SWV) technique, this biosensor can detect from 0.001 to 100 ng/mL of MERS-CoV (with the LOD = 0.4 pg/mL) in a short period (20 min). Similarly, AgNPs were used for detecting the influenza virus because AgNPs are a type of metal nanoparticle that exhibits good electrochemical behavior, including long-term stability.

However, using only AgNPs to label the biomolecules causes low sensitive results. Huang et al. enhanced the signal by developing a biosensor for detecting the influenza virus using nanocomposite composed of antibodies, AgNPs, graphene, and chitosan. In their research, the nanocomposite was used as a signal probe to increase an electrochemical signal by enlarging the surface area and immobilization rate of signal-inducing materials. In the presence of influenza viruses, the signal probe was immobilized on the electrode surface by a sandwich-type immuno-reaction consisting of antibodies on the signal probe, viruses, and antibodies on the electrode. The biosensor analyzed the electrochemical signal of the signal probe and detected the influenza virus in the range of 1.6 × 10^−3^-6 ng/mL with a low detection limit of 1.6 pg/mL using linear sweep voltammetry (LSV), which is an electrochemical analysis method (Huang et al., 2016).

both AuNPs and AgNPs amplify signals with similar properties in electrochemical measurements, but they have some differences. As an example of loading DNA on the surface of nanoparticles, AuNPs are well-separated with both low and saturated loading of DNA. In contrast, AgNP exhibited a substantial agglomeration, with both low and high loading of DNA (Malecka et al., 2021). Furthermore, Malecka et al. reported that AgNPs required lower DNA loading than AuNPs for the most accurate DNA detection. Accordingly, AgNPs can be a candidate as a biosensor component given their cost-effectiveness, whereas AuNPs can be preferred for their easy handling and high stability. Consequently, if AuNPs and AgNPs are used for electrochemical sensors, the material must be determined according to the needs and characteristics.

Of the noble metal nanoparticles, PtNPs have been used in probes of electrochemical biosensors because of their broad range of electrocatalytic activity, such as in oxidation reactions (Li and Baek, 2020). For instance, Yang et al. used PtNPs' catalytic oxidation of 1-naphthol (1-NP) for detecting the influenza virus using a sandwich immunoassay (Yang et al., 2016). Briefly, they developed PtNPs and hemin modified porous ZnO sphere (Pt-pZnO-hemin). The nanocomposite was functionalized with alkaline phosphatase (ALP) and anti-influenza-antibody (Ab/ALP/Pt-pZnO-hemin). When the influenza existed, the Ab/ALP/Pt-pZnO-hemin was immobilized on the surface of antibody/Au modified electrode via the sandwich structure. The immobilized nanocomposite displayed cascade reactions and demostrated electrochemical signal. First, ALP catalyzed the conversion of 1-naphthyl acid phosphate (p-NPP) into an electrochemical active 1-naphthol (p-NP). Pt-hemin catalyzed the conversion of p-NP to 2-hyodroxy-1,4-naphthoquinone in the presence of H_2_O_2_. From these reactions, electrochemical signal was generated and amplified by cascade reactions according to the amount of immobilized Ab/ALP/Pt-pZnO-hemin on the electrode via influenza. By using the biosensor composed of Ab/ALP/Pt-pZnO-hemin, an amplified electrochemical signal from catalytic oxidation of 1-NP by PtNPs was observed, and the influenza virus was detected from a range of 1.0 × 10^−3^-60 ng/mL (LOD = 7.6 × 10^−1^ pg/mL) (Figure 1B).

## Optical Biosensors For Virus Detection

Optical phenomena including colorimetry, fluorescence, and SERS have been used in various fields due to advantages such as relatively less complicated instrumentation, ease of use, and cost-effectiveness. Because these advantages are suitable for a point-of-care test (POCT) system, many researchers used optical phenomena for the biosensor to detect target biomolecules (Lee et al., 2019; Maddali et al., 2020). Accordingly, the optical properties of noble metal nanoparticles are desirable in the field of biosensors.

In optical biosensors, the specific colors of noble nanoparticles have been widely used because their colors can be detected with the naked eye (Pham et al., 2020). Also, low-cost instrumentation, such as a smartphone camera, is required for more sensitive detection. AuNPs, AgNPs, and PtNPs have red, brown, dark brown colors, respectively. However, the colors of noble metal nanoparticles are not always constant. The colors of particles depend on the particle size or agglomeration of particles. For example, the color of the AuNPs changes from red to purple when the AuNPs are agglomerated. The phenomenon can be applied for detecting SARS-CoV-2 using antibodies (spike/membrane/envelope antibodies of SARS-CoV-2)-functionalized AuNPs (f-AuNPs) (Ventura et al., 2020). When SARS-CoV-2 interacted with f-AuNPs as an antigen-antibody reaction, f-AuNPs were agglomerated on the surface of the virus, resulting in a color change. This colorimetric detection method detected extremely low viral load with the detection limit approaching that of real-time PCR.

Although most studies use AuNPs that change color distinctly, the color change of AgNPs according to the agglomeration is also applied to detecting DNA because AgNPs exhibited improved colorimetric sensitivity, resulting from a higher extinction coefficient than AuNPs. Teengam et al. used pyrrolidinyl peptide nucleic acid (acpcPNA)-induced AgNPs aggregation to enable the colorimetric biosensors to detect target DNA extracted from MERS-CoV (Teengam et al., 2017). Without the target DNA, acpcPNA illustrates a positive charge that causes it to agglomerate with negative charged AgNPs. However, in the presence of the target DNA, the formation of anionic DNA–acpcPNA complex produced a negative charge. As a result of electrostatic repulsion, AgNPs produced a detectable color change according to the target DNA. With the proposed method, the MERS-CoV target DNA could be detected with detection limits of 1.53 nM (Figure 1C).

In the development of biosensors, the color of noble metal nanoparticles has been used not only to change color but also to use color itself as a probe. However, biosensors, which used the color of the noble metal nanoparticles themselves, have limited detection sensitivity. PtNPs have a dark brown color with relatively high visual contrast, but the color of PtNPs is not as vivid as AuNPs. Therefore, there is a need to enhance the color signal of noble metal nanoparticles for the sensitive detection of analytes.

Matsumura et al. enhanced the color signal by developing signal-enhanced Pt-latex nanoparticles to detect the influenza virus and applied them in an immunochromatographic test (ICT). The color of PtNPs can be enhanced by a latex organic nanocomposite, with a more vivid color than PtNPs. Consequently, the noble metal-organic nanocomposite-based biosensor exhibited higher sensitivity than the bare noble metal nanoparticle-based biosensor (Matsumura et al., 2018).

Noble metal nanoparticles also exhibit a localized SPR (LSPR) property induced by the collective oscillation of electrons in resonance with the incident light frequency. Because of the LSPR property, the noble metal nanoparticles have been used for the signal enhancement of fluorescent inorganic quantum dots (QD) influenced by the adjacent metallic nanoparticles. Due to the easy fabrication process, high sensitivity, and high fluorescence enhancing effect, noble metal nanoparticle-based LSPR-fluorescence-biosensor has been developed in virus sensing fields.

For example, Nasrin et al. used the surface plasmon effect of AuNPs for the fluorescent detection of the influenza virus (Nasrin et al., 2020). When the distance between the QD and AuNP was the optimal length of the linker peptide, AuNPs could enhance the fluorescent properties of QDs due to the surface plasmon effect of AuNPs. When the virus was bound to the linker peptides, the fluorescence activity was quenched by steric hindrance on the LSPR behavior. Nasrin et al. used this method and detected the influenza virus in a range of 10^−14^-10^−9^ g/mL with a detection limit of 1.7 × 10^−2^ pg/mL (Figure 1D).

SERS is a powerful tool for detecting viruses because it enables label-free, highly sensitive detection. Noble metal nanomaterials have been used as a SERS-active probe because of their unique SPR property inducing substantial SERS enhancement (Lee and Choi, 2019). Maneeprakorn et al. used star-shaped AuNPs (AuNS), which had multi-arms and surface roughness for detecting the influenza A virus (Maneeprakorn et al., 2016). Because the feature of AuNS enables high SERS performance due to their tunable surface plasmon and multiple sharp branches, AuNS combined with Raman active molecule (4-amino thiophenol) and influenza A nucleoprotein specific antibody nanoparticle was used as a SERS signal reporter and detection probe. Based on the probe, a sensitive SERS-based lateral flow immune-chromatographic test system was developed. This biosensor demonstrated excellent sensitivity in detecting the influenza A virus with an LOD of 6.7 ng/mL.

Although AgNPs have less chemical stability than AuNPs, AgNPs are more plasmonically active than AuNPs and produced a strong SERS signal (Hassan et al., 2021). Therefore, in SERS-based biosensing techniques, many researchers have applied AgNPs for the sensitive detection of targets. Liu et al. developed a SERS-based lateral flow immunoassay (LFIA) to detect the SARS-CoV-2 antibodies (IgM/IgG) using SiO_2_@AgNPs (Liu et al., 2021). In the research, the Ag shell on SiO_2_ core (SiO_2_@AgNP) coated with Raman dye was fabricated to obtain a strong signal reporter, and SiO_2_@AgNPs exhibited high stability and fine SERS signals because of its high monodispersity. After the SARS-CoV-2 spike protein was modified on SiO_2_@AgNPs, the NPs were simultaneously captured on two zones at the LFIA biosensor—anti-human IgM and IgG antibodies immobilized, respectively—using antibody-antigen reactions. Based on the analysis of SERS intensity of two zones, two zones with anti-human IgM/IgG antibodies on the biosensor demonstrated excellent SERS intensity via SiO_2_@AgNPs in the presence of SARS-CoV-2 antibodies.

Table 1 summarizes noble metal nanoparticle-based biosensors for detecting viruses, particularly those causing respiratory problems. Noble metal nanoparticles are highly attractive sensing materials for detecting viruses. Given the unique properties of noble metal nanoparticles, they have the potential to develop further in the field of biosensing technologies.

**Table 1:** Noble metal nanomaterials and their application to virus biosensors.

**Core noble metal nanomaterials**	**Techniques**	**Target virus**	**Analyte**	**Limit of detection**	**References**
AuNPs	Electrochemical (DPV)	SARS-CoV-2	Nucleic acids (RNA)	200 copies/mL	Zhao et al., 2021
	Electrochemical (DPV)	SARS-CoV-2	Protein	7.8 × 10^−1^ pg/mL	Mahari et al., 2020
	Electrochemical (SWV)	MERS-CoV	Protein	0.4 pg/mL	Layqah and Eissa, 2019
	Optical (Fluorescence)	Influenza virus	Protein	1.7 × 10^−2^ pg/mL	Nasrin et al., 2020
	Optical (SERS)	Influenza virus	Protein	6.7 × 10^3^ pg/mL	Maneeprakorn et al., 2016
AgNPs	Electrochemical (LSV)	Influenza virus	Protein	1.6 pg/mL	Huang et al., 2016
	Optical (Colorimetry)	MERS-CoV	Nucleic acids (DNA)	1.53 nM	Teengam et al., 2017
	Optical (SERS)	SARS-CoV	Protein	1 pg/mL	Liu et al., 2021
PtNPs	Electrochemical (DPV)	Influenza virus	Protein	7.6 × 10^−1^ pg/mL	Yang et al., 2016
	Optical (Colorimetry)	Influenza virus	Protein	2.5 × 10^−2^ HAU/mL	Matsumura et al., 2018

## Concluding Remarks And Future Perspectives

This mini-review summarizes noble metal nanomaterials with unique properties and their applicability in developing biosensors for detecting respiratory illness-inducing viruses. The fabricated biosensors composed of noble metal nanomaterials demonstrated excellent biosensing abilities, including high sensitivity, selectivity, and stability. The detection of previous respiratory illness-inducing viruses that caused epidemics and attempts to detect SARS-CoV-2 using developed biosensors were demonstrated. Even though there are no specific treatments for SARS-CoV-2 thus far, early diagnosis with rapid detection using the developed biosensors could decrease the contagion and fatalities associated with SARS-CoV-2. the biosensing platform comprising noble metal nanomaterials can be used to make other biosensors by exchanging the probe of biosensors. This suggests the immediate applicability of well-developed biosensors to newly discovered viruses and their variants if their targets and probes are studied.

Noble metal nanomaterials have unique properties for accurate and sensitive biosensing. However, there are limitations associated with their application. First, there are difficulties in synthesizing the noble metal nanomaterials, with morphology control of the noble metal nanomaterials for the application dependent on the fabrication method. For instance, with soft and nanoimprint lithography, ultra-small nanoparticles can be synthesized with a simple process. However, lithography techniques are not cost-effective and are challenging during the large-scale production of densely packed nanostructures (Khan et al., 2019). To mass produce the biosensors for virus detection, this disadvantage would be an obstruction and would slow down biosensor development. For example, most of the recently commercialized biosensors for COVID-19 are paper-based biosensors (Choi, 2020). Considering the urgency of COVID-19, paper-based biosensors with user-friendly, cost-effective, and simple fabrication methods are preferred over other types of biosensors.

Furthermore, some noble metal nanomaterials (e.g., silver) exhibit toxicity and low biocompatibility with biosamples—a significant obstacle for the further application of biosensors composed of the noble metal nanoparticles, such as *in vivo* applications. The hybridization of noble metal nanomaterials with other components such as carbon-based materials and other 2D materials may help overcome this limitation. For instance, carbon-based materials are also suitable for use in biosensors due to their biocompatibility and excellent electrical and optical abilities (Wu et al., 2011; Nguyen et al., 2020; Zhang Y. et al., 2020). Other 2D materials, such as transition metal dichalcogenide materials, can enhance biosensor performance due to their electrical properties (Yoon et al., 2019; Zhao P. et al., 2020). Furthermore, enhancing sensing signal through material hybridization can increase the sensitivity of biosensors using only a small amount of essential biomaterials—the primary cost burden for large-scale production.

Thus far, nanohybrids composed of noble metal nanomaterials and other biosensor components have been developed to improve their sensing performance and biocompatibility. Noble metal nanomaterials are critical core materials for fabricating biosensors that detect not only viruses but also other target biocomponents using various sensing techniques. Furthermore, there is a developmental potential in broad aspects for the noble metal nanomaterials using various applications, such as the hybridization of noble metal nanomaterials with other materials or controlling the structure of noble metal nanomaterials. We believe that improvements in biosensors comprising noble metal nanomaterials will enable the early diagnosis of major new diseases, including respiratory illnesses causing an epidemic or a pandemic, more efficiently and help promote wellness globally.

## Author Contributions

HC and M-JL conceived the work, discussed the content, and drafted the manuscript. SL and T-HK were responsible for revising it. T-HK and B-KO critically reviewed, edited, and finalized the manuscript for submission.

## Conflict of Interest

SL was employed by the company Uniance Gene Inc. The remaining authors declare that the research was conducted in the absence of any commercial or financial relationships that could be construed as a potential conflict of interest.
